# A Crude 1-DNJ Extract from Home Made Bombyx Batryticatus Inhibits Diabetic Cardiomyopathy-Associated Fibrosis in db/db Mice and Reduces Protein *N*-Glycosylation Levels

**DOI:** 10.3390/ijms19061699

**Published:** 2018-06-07

**Authors:** Qing Zhao, Tian Zhu Jia, Qi Chen Cao, Fang Tian, Wan Tao Ying

**Affiliations:** 1 The Key Laboratory of Chinese Materia Medica Processing Principle Analysis of the State Administration of Traditional Chinese Medicine, Pharmaceutical College of Liaoning Traditional Chinese Medicine University, Chinese Materia Medica Processing Engineering Technology Research Center of Liaoning Province, Dalian 110060, China; wshxr2003@163.com; 2 Chinese Materia Medica Department, Traditional Chinese Medicine College of Hebei University, Baoding 071000, China; 3 Beijing Institute of Lifeomics, Beijing Proteome Research Center, Beijing 102206, China; finninn@163.com; 4 Tianjin Institute of Industrial Biotechnology, Chinese Academy of Sciences, Tianjin 300308, China; cao_qc@tib.cas.cn

**Keywords:** 1-DNJ, diabetic cardiomyopathy, fibrosis, *N*-glycosylation, α-1,6-fucosylation

## Abstract

The traditional Chinese drug Bombyx Batryticatus (BB), which is also named the white stiff silkworm, has been widely used in Chinese clinics for thousands of years. It is famous for its antispasmodic and blood circulation-promoting effects. Cardiomyocyte hypertrophy, interstitial cell hyperplasia, and myocardial fibrosis are closely related to the *N*-glycosylation of key proteins. To examine the alterations of *N*-glycosylation that occur in diabetic myocardium during the early stage of the disease, and to clarify the therapeutic effect of 1-Deoxynojirimycin (1-DNJ) extracted from BB, we used the db/db (diabetic) mouse model and an approach based on hydrophilic chromatography solid-phase extraction integrated with an liquid Chromatograph Mass Spectrometer (LC-MS) identification strategy to perform a site-specific *N*-glycosylation analysis of left ventricular cardiomyocyte proteins. Advanced glycation end products (AGEs), hydroxyproline, connective tissue growth factor (CTGF), and other serum biochemical indicators were measured with enzyme-linked immunosorbent assays (ELISA). In addition, the α-1,6-fucosylation of *N*-glycans was profiled with lens culinaris agglutinin (LCA) lectin blots and fluorescein isothiocyanate (FITC)-labelled lectin affinity histochemistry. The results indicated that 1-DNJ administration obviously downregulated myocardium protein *N*-glycosylation in db/db mice. The expression levels of serum indicators and fibrosis-related cytokines were reduced significantly by 1-DNJ in a dose-dependent manner. The glycan α-1,6-fucosylation level of the db/db mouse myocardium was elevated, and the intervention effect of 1-DNJ administration on *N*-glycan α-1,6-fucosylation was significant. To verify this result, the well-known transforming growth factor-β (TGF-β)/Smad2/3 pathway was selected, and core α-1,6-fucosylated TGF-β receptor II (TGFR-βII) was analysed semi-quantitatively with western blotting. The result supported the conclusions obtained from LCA lectin affinity histochemistry and lectin blot analysis. The expression level of α-1,6-fucosyltransferase (FUT8) mRNA was also detected, and the results showed that 1-DNJ administration did not cause obvious inhibitory effects on FUT8 expression. Therefore, the mechanism of 1-DNJ for relieving diabetic cardiomyopathy (DCM)-associated fibrosis can be concluded as the inhibition of *N*-acetylglucosamine (*N*-GlcNAc) formation and the reduction of substrate concentration.

## 1. Introduction

Diabetes mellitus (DM) is a common type of endocrine and metabolic disease. It is not only characterized by long-term chronic plasma hyperglycemia, but is also accompanied by a variety of deadly complications [1,2]. Among these, cardiovascular complications are some of the most harmful, because of their high morbidity and mortality [3,4]. Type 2 diabetes is associated with a cardiac syndrome called diabetic cardiomyopathy (DCM), which is characterized by left ventricular contractile dysfunction [5], cardiac hypertrophy [6], interstitial collagen accumulation [7], and fibrosis [8,9]. The pathogenesis of DCM is extremely complicated, and the underlying mechanisms require further study; however, the signalling disturbances associated with cardiac insulin resistance have been elucidated [10]. Since glucose is involved in the signalling processes of myocardial structural and functional modelling [11], the heart is subjected to various glucose-mediated challenges, including the suppression of glucose oxidation, glycogen accumulation, and the elevation of protein glycation levels [12].

Evidence suggests that non-enzymatic glycation of collagen occurs in the myocardial interstitium and microvascular wall to form advanced glycation end products (AGEs) [13], which mainly bind to the receptor for AGEs (RAGE) to induce fibroblast proliferation [14]. Sustained hyperglycemia can lead to this process, which can then promote the release of some cytokines and growth factors, such as connective tissue growth factor (CTGF) and transforming growth factor-β (TGF-β) [15]; the massive release of CTGF during the course of long-term diabetes will cause strong expression of extracellular matrix (ECM) components [16,17], which can promote cell hypertrophy and interstitial fibrosis, leading to altered cardiac function. In addition, TGF-β can induce cell hypertrophy and mesangial matrix expansion through a series of signalling interactions with receptors [18].

It has been shown that the hexosamine biosynthesis pathway (HBP) contributes to diabetes-associated complications in many tissues of DM patients [19]. Inevitably, high levels of glucose must be enzymatically processed by the HBP, leading to an excess of circulating *N*-acetylglucosamine (*N*-GlcNAc) [20]. 

In recent years, asparagine (N)-linked glycosylation has emerged as an essential indicator for evaluating the endocrine and metabolic status of DM patients, and some *N*-glycoproteins have been used as biomarkers in clinical diagnosis. Previously, an increase in the α-1,3-fucosylation of α1-acid glycoprotein (AGP) was found in the serum and urine of DM patients [21,22]. In addition, there is emerging evidence for a role of *N*-glycosylation in the pathogenesis of voltage-gated calcium (Ca^2+^) channels (VGCCs); for example, the altered glycosylation of T-Type Ca^2+^ Channel (Cav3.2) may contribute to an enhanced T-type calcium current in dorsal root ganglion (DRG) neurons during diabetes [23]. Serum glycol proteomic analysis indicated that compared with that of normal mice, the serum of db/db mice contained more *N*-glycoproteins with α-1,6-fucose glycan structures, and the same result was also found in serum from patients with type 2 diabetes [24]. Moreover, in the well-known TGF-β/Smad2/3 pathway, which is related to cell hypertrophy and mesangial matrix expansion, both the TGFβ-I receptor and the TGFβ-II receptor are *N*-glycoproteins, and have been shown to be modified with a core α-1,6-fucose. If this modification is blocked, the subsequent steps of TGF-β signalling and the phosphorylation of regulatory Smad2 and Smad3 (R-Smads) are also terminated [25]. At present, TGF-β has been verified as one of the molecular mediators involved in the progression of fibrosis in DCM [26].

All the studies mentioned above have clarified the striking changes in the structure and amount of *N*-glycan targeting in some specific glycoproteins, and some findings have had important implications for clinical treatment and the development of new drugs. However, a comprehensive N-linked glycosylation proteomic study has not yet been performed. In addition, a number of reports highlight alterations of N-linked glycosylation, but none of those changes are correlated with DCM. Further, these results were obtained in advanced stages of this disease, but less is known about the direct impact of hyperglycemia on glycan biosynthetic processes and the site-specific glyco-decoration of cellular proteins during the early stage of the disease. Therefore, a detailed investigation of the differences in the *N*-glycosylated proteome among DCM tissues, normal tissues, and myocardium tissues treated with drugs is needed.

To date, there is no specific therapy available for the development of pre-diabetic cardiomyopathy; apart from blood glucose level control, the main drug interventions for DCM include angiotensin-converting enzyme inhibitors [27], statins [28], and endothelin receptor inhibitors [29]. Therefore, there is an urgent need to develop new drugs to treat DCM in this specific context.

1-Deoxynojirimycin (1-DNJ) is a kind of piperidine alkaloid, and its chemical name is (2*R*,3*R*,4*R*,5*S*)-2-hydroxymethyl-piperidine-3,4,5-triol. This alkaloid is synthesized in a relatively large amount in the leaves and root bark of mulberry, compared to other plants [30]. In addition, silkworms can specifically enrich 1-DNJ [31], so some scholars have used silkworm powder to treat diabetes with good results [32]. The stiff silkworm, which is named Bombyx Batryticatus (BB), refers to dead silkworm larvae infected with *Beauveria bassiana* (Bals.) Vuill. [33]. This traditional drug has been used widely in clinics in China. Compared with that of the silkworm, the annual output of BB is greater, and the price is lower. Therefore, BB is a better raw material for extracting 1-DNJ. Many reports showed that 1-DNJ is an α-glucosidase inhibitor [34]; more importantly, it has been reported that N-linked complex oligosaccharide formation in cell lines could be inhibited by 1-DNJ [35].

We therefore hypothesized in this study that the reduction of *N*-GlcNAc may be a mechanism by which 1-DNJ could relieve cardiomyopathy and fibrosis caused by diabetes in the type 2 diabetic mouse heart. However, since these interactions have not been studied in the heart, the secondary purpose of this study was to characterize the effects of 1-DNJ extract administration on α-linked fucose glycosylation. The expression of the TGF-β/Smad2/3 complex, which is regulated by the core α-1,6-fucosylation of TGFR-β, was used to verify this hypothesis. We used five-week-old db/db mice, which were gavaged with the 1-DNJ extract from BB for six weeks, to investigate the early signalling mechanisms during the cardiac hypertrophy and fibrosis process. Our work provided a new understanding of how 1-DNJ protects against DCM, and these results should be considered in future therapeutic strategies.

## 2. Results

### 2.1. 1-Deoxynojirimycin Enrichment and Purity Test

**Figure 1:**
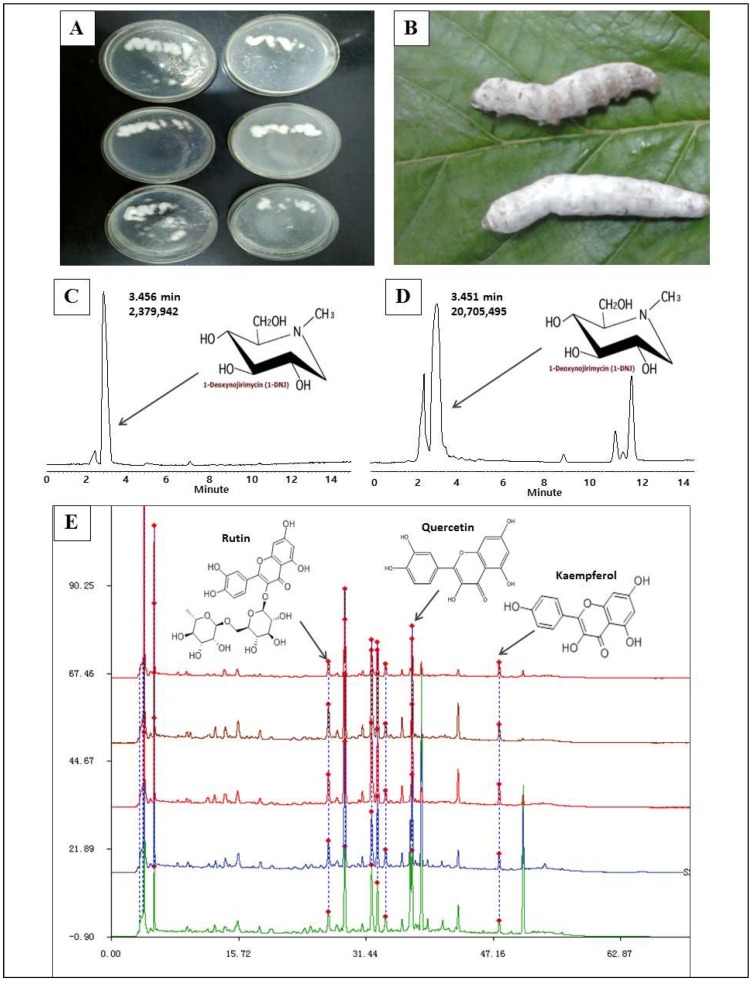
BB preparation and 1-Deoxynojirimycin (1-DNJ) extract purity detection with High Performance Liquid Chromatography (HPLC). **A**: The subcultured standard strains of *Beauveria bassiana.* **B**: Infected silkworms became stiff and white. **C**: Liquid chromatogram of the 1-DNJ standard. **D**: Liquid chromatogram of 1-DNJ extracted form Bombyx Batryticatus. **E**: Several flavonoids were identified and assayed in the 1-DNJ crude extracts by HPLC.

Because of its high yield and relatively low price, BB is an ideal material for the extraction of 1-DNJ. However, the BB that can be purchased commercially is sometimes contaminated with *Aspergillus flavus* [36]. To ensure the safety of the materials, we prepared BB ourselves. The quality of the BB was fine; from Figure 1, we can see that the BB bodies were stout and wrapped with a thick layer of white mycelium (Figure 1B). From 5.0 kg of BB, we obtained 19.72 g of 1-DNJ. The purity was determined with High Performance Liquid Chromatography (HPLC), and the content was 87.2% (Figure 1C,D). In addition, some flavonoids, such as rutin, quercetin, and kaempferol, were also detected, with contents of 2.8%, 6.5%, and 3.5%, respectively (Figure 1E,F). 

### 2.2. Effects of 1-Deoxynojirimycin on Clinical Biochemistry Indicators in db/db Mice

**Figure 2:**
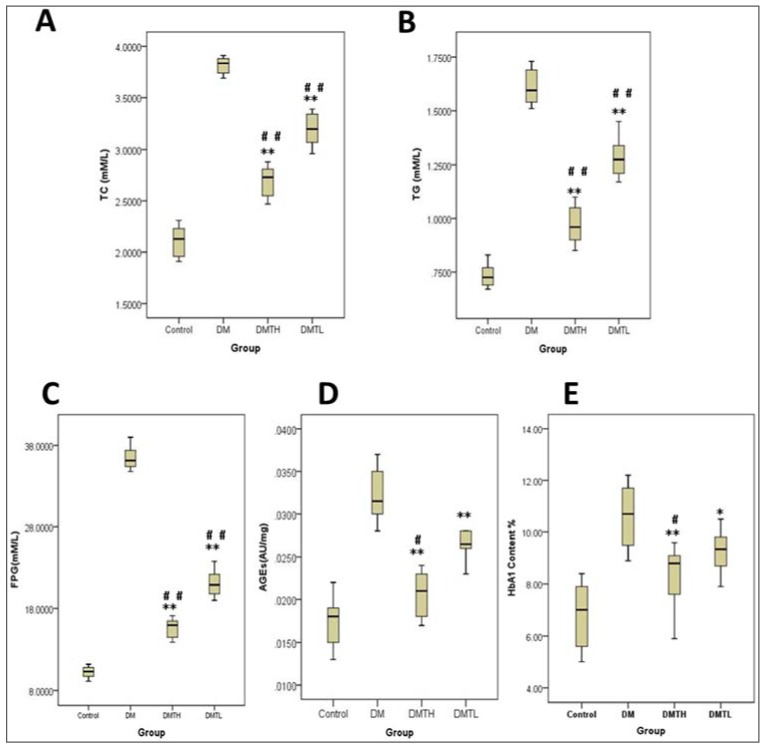
Box plot analysis of the effects of 1-DNJ on some clinical indicators in diabetic (db/db) mice with IBM-SPSS 19.0. **A**: Effect of 1-DNJ on total serum cholesterol of mice. **B**: Effect of 1-DNJ on fast plasma glucose of mice. **C**: Effect of 1-DNJ on the serum triglyceride of mice. **D**: Effect of 1-DNJ on advanced glycation end products (AGEs) of mice. **E**: Effect of 1-DNJ on glycated hemoglobin of mice. All the above data were analyzed with the one-way ANOVA method ($\bar{X}\pm S.$ vs. Control: ** represents *p* < 0.01; * represents *p* < 0.05; vs. DM: ## represents *p* < 0.01; # represents *p* < 0.05).

The noted hypoglycemic effect of 1-DNJ has been reported for many years. However, to further assess the effects of 1-DNJ on lipid metabolism and glycated protein formation in db/db mice during the early stage of the disease, we also inspected the expression levels of some biochemical indicators in the serum of the animals. From Figure 2, we found that total cholesterol (TC), triglycerides (TG), fasting blood glucose (FBG), glycated hemoglobin (HbA1), and AGEs were all expressed at the highest levels in the DM groups. In the 1-DNJ-treated groups, the levels of these indicators decreased significantly (*p* < 0.05), and the positive effects exhibited dose dependence; in other words, the inhibitory effects observed in the DM high-dose therapy (DMTH) group were much stronger than those of the DM low-dose therapy (DMTL) group. Interestingly, the expression patterns of all the indicators were similar in the four groups, and the results showed that 1-DNJ can regulate lipid metabolism and inhibit glycated protein formation in db/db mice.

### 2.3. Myocardial Histomorphometry

**Figure 3:**
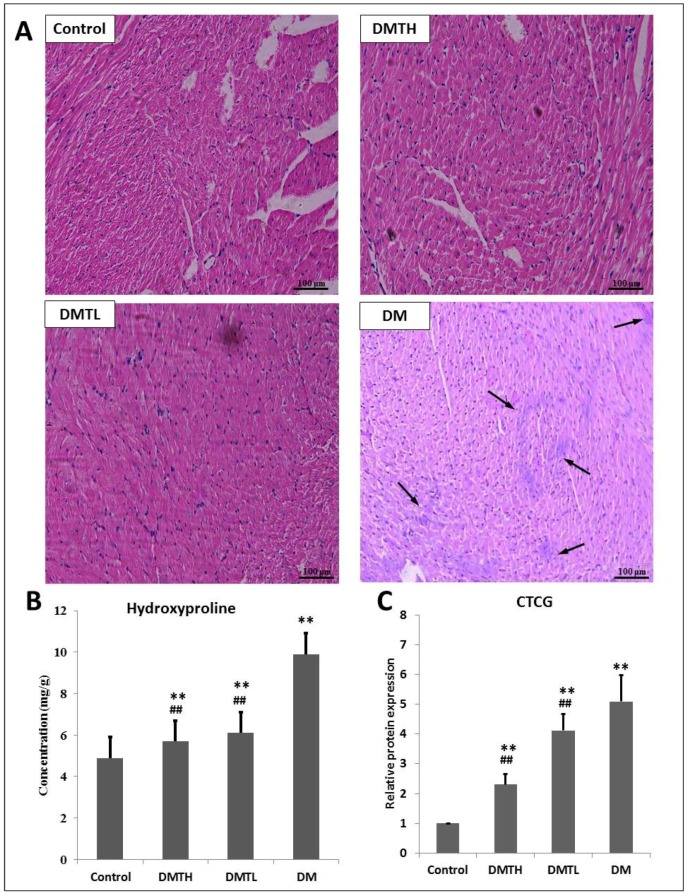
Left ventricular muscle histopathology and expression levels of key biomarkers in the myocardium of four groups of mice. **A**: Histopathological sections were stained with hematoxylin and eosin (×100). **B**: Hydroxyproline expression measurement with ELISA kits. **C**: Connective tissue growth factor (CTCG) expression measurement with ELISA kits. ($\bar{X}\pm S.$ vs. Control: ** represented *p* < 0.01; * represents *p* < 0.05; vs. DM: ## represents *p* < 0.01; # represents *p* < 0.05). Hydroxyproline and CTCG expression comparison was analyzed with one-way ANOVA method in IBM-SPSS 19.0.

To better assay the myocardial morphological changes that occur in the early stage of disease in db/db mice, and to detect the intervention effects of 1-DNJ, we used mouse left ventricular paraffin sections and haematoxylin and eosin (HE) staining for histopathological examination (Figure 3). In histopathology sections from the DM group, we can see that coagulation necrosis occurred in the cardiomyocytes, and the necrotic cardiomyocytes were swollen, exhibited pyknosis, or appeared to be dissolved. The sarcoplasm was dissolved, and the stripes disappeared. Myocardial fibres in the left ventricular endocardium disappeared, and scars formed between the tiny myocardial fibres (indicated by the arrows). In the DMTH group, the left ventricular cardiomyocytes were relatively intact, and no scar formation was observed, but individual cell nuclei exhibited ablation. The myocardial tissue of mice in the DMTL group presented with some necrosis of cardiomyocytes, and some cells had fuzzy edges with a shrinking nucleus. Moreover, minor disappearance of myocardial stripes can also be observed in the slice.

### 2.4. Regulatory Effect on the Expression Levels of Connective Tissue Growth Factor and Hydroxyproline in Myocardial Tissues

A large number of cross-sectional studies and prospective studies have shown that CTGF is closely related to myocardial fibrosis [37]. In addition, hydroxyproline mainly exists in collagen. When myocardial fibrosis occurs, the major component of the myocardium is collagen fibres, and hydroxyproline is unique to collagen fibres [38]. Therefore, the amount of hydroxyproline can be measured to monitor the number of collagen fibres and reflect the degree of myocardial fibrosis. Therefore, we quantified the expression levels of these two indicators with enzyme linked immunosorbent assays (ELISA) kits. From Figure 3, we can see that compared with those of the control group, the levels of CTGF and hydroxyproline were both upregulated significantly (*p* < 0.01) in the DM group. 1-DNJ administration induced an obvious downregulation of CTGF (*p* < 0.01) and hydroxyproline (*p* < 0.01) in db/db mice and showed a dose-dependent relationship.

### 2.5. Site-Specific N-Glycosylated Proteome Identification and Motif Analysis 

**Figure 4:**
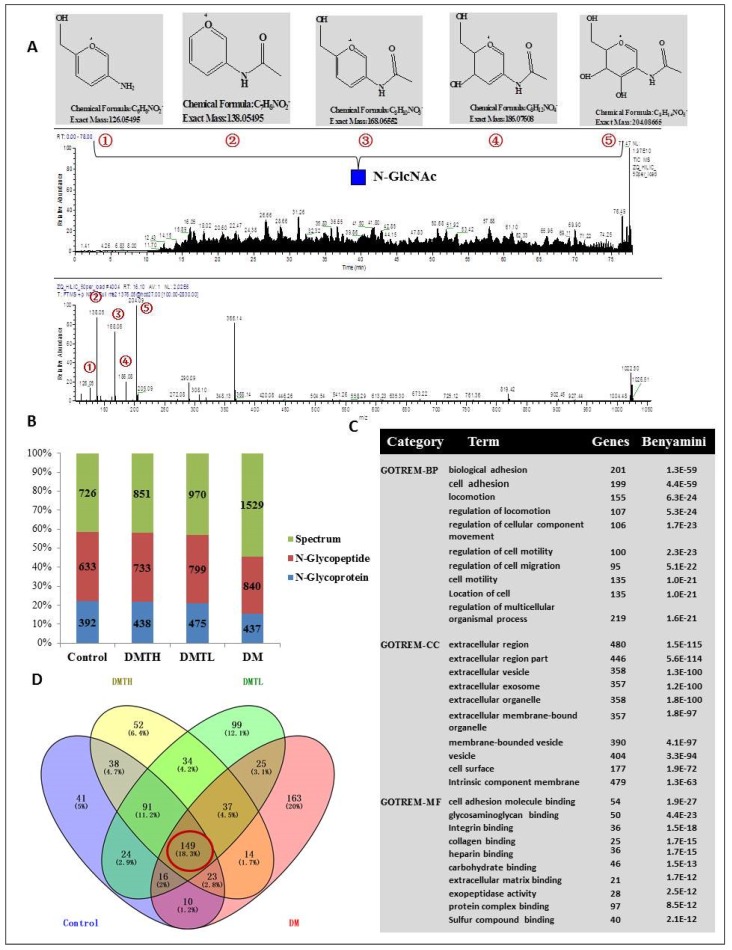
Basic bioinformatics analysis of the *N*-glycosylation profiles of mouse myocardial tissue based on hydrophilic chromatography enrichment and LC-MS/MS technology. **A**: Spectrum of an N-linked peptide total ion chromatogram (TIC) in this analysis. ①~⑤ represent the oxonium ion fragmentation of *N*-acetylglucosamine (*N*-GlcNAc). **B**: Mass spectrometry-based *N*-glycosylation matched results obtained from a database search with samples from the four groups (FDR < 0.01). **C**: Gene functional annotation classification with “the Database for Annotation, Visualization and Integrated Discovery “(DAVID) analysis of the *N*-glycoproteins btained from these four groups. **D**: Overlapping relationships of the identified *N*-glycoproteins from the four groups (analyzed by Venny2.1.0).

To further evaluate the *N*-glycosylation of db/db mouse myocardial proteins and explore the expression differences between the groups that were treated with or without 1-DNJ, we used hydrophilic solid-phase extraction and biological mass spectrometry identification to perform site-specific *N*-glycosylated proteome analysis (Figure 4A). To improve the accuracy of identification, we extracted the intensity of *N*-glycosylated peptides based on a Perl program, combined with the official Xcalibur software (Thermo Scientific, Waltham, MA, USA). Peptides with an intensity of “null” were removed. The corrected results are shown below. For the control group, 633 non-redundant *N*-glycosylated peptides with 664 sites were identified from 726 spectrograms, which corresponded to 392 protein groups. Similarly, 733 non-redundant *N*-glycosylated peptides with 762 sites from 851 spectrograms were identified as corresponding to 475 protein groups. For the DMTH group, 799 non-redundant *N*-glycosylated peptides with 832 sites were identified from 970 spectrograms, and they corresponded to 475 protein groups. Notably, 839 non-redundant *N*-glycosylated peptides, with 872 sites in 1630 spectrograms corresponding to 437 protein groups, were identified in the DM group (Figure 4B). Venny2.1.0-based (Juan Carlos Oliveros Bioinformatics, Madrid, Spain) overlap analysis revealed that only 127 identified N-linked proteins were co-expressed in the four groups (Figure 4D). Subcellular location analysis revealed that most of the *N*-glycosylated proteins were located in the ECM, plasma membrane, or membrane-bound organelles. In addition, there were 96 proteins with a molecular function annotated as “protein binding”, such as a Na^+^/K^+^-transporting polypeptide, a CD48 antigen, an Fc IgG low-affinity receptor, a cell adhesion molecule, and an activated leukocyte cell adhesion molecule. From Figure 4, we could see the unique protein distributions of the myocardial tissues from the four groups of mice. There were 41 unique proteins related to cell adhesion, metabolic processes, and differentiation in the control group, and there were 51 unique proteins related to cell growth, cell adhesion, and metabolic processes in the DMTH group. There were 98 unique proteins related to glycoprotein metabolic processes, carbohydrate derivative metabolic processes, and ECM organization in the DMTL group, and there were 163 unique proteins related to cell adhesion, angiogenesis, regulation of cell motility, and regulation of cell migration in the DM group. We also performed gene ontology analysis, and the results indicated that the involved biological processes were described by many categories; according to the Benjamini and Hochberg corrected *p*-value, we chose the top 10 reliable classification categories (Benjamini coefficient between 1.3 × 10^−59^ and 1.6 × 10^−^^21^), such as biological adhesion, cell adhesion, locomotion, regulation of locomotion, and regulation of cellular components. The cellular components (Benjamini coefficient between 1.5 × 10^−115^ and 1.3 × 10^−^^63^) corresponded to the extracellular region, extracellular vesicles, membrane-bound vesicles, cell surface components intrinsic to the membrane, and other components. Similarly, the molecular functions of these proteins (Benjamini coefficient between 1.9 × 10^−27^ and 2.1 × 10^−12^) could be summarized as cell adhesion molecule binding, glycosaminoglycan binding, integrin binding, heparin binding, carbohydrate binding, and others (Figure 4C).

**Figure 5:**
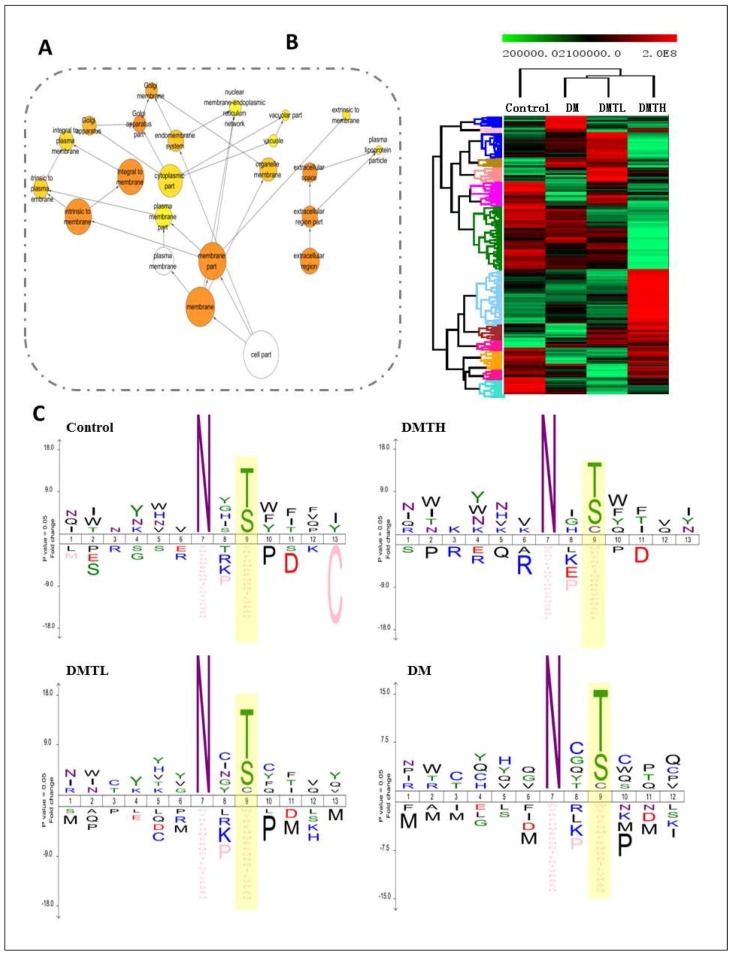
Subcellular location, gene symbol cluster, and *N*-glycosylation motif analysis. **A**: Subcellular locations of identified proteins with *N*-glycosylation. **B**: Cluster analysis of the proteins from the four groups (analyzed by MeV-4.9.0 software). **C**: Motifs of the identified *N*-glycoproteins based on iceLogo program analysis.

The gene symbol cluster analysis in Figure 5 shows that the relationship between the DM group and the DMTL group was stronger than the relationship between any other two groups (Figure 5B). The network relationship analysis indicated that the subcellular locations of the identified *N*-glycosylated proteins were extracted as the membrane region and the extracellular region. This result suggested that the hyperglycemic state may promote the *N*-glycosylation of proteins of the plasma membrane, interstitial cells, and the ECM (Figure 5A). *N*-glycosylation site analysis was performed with the iceLogo program (http://www.twosamplelogo.org/). When setting the parameters, we used asparagine as the centre, and six amino acids were included in the forward and reverse directions. The presence of the amino acid sequence N-X-T at the *N*-glycosylation site generates a stronger motif than does the presence of the N-X-S. More importantly, the glycosylated site of the control group was present as N-X-T/S, while the *N*-glycosylated sites of the other three groups had the amino acid sequence N-X-T/S/C (Figure 5C).

**Figure 6:**
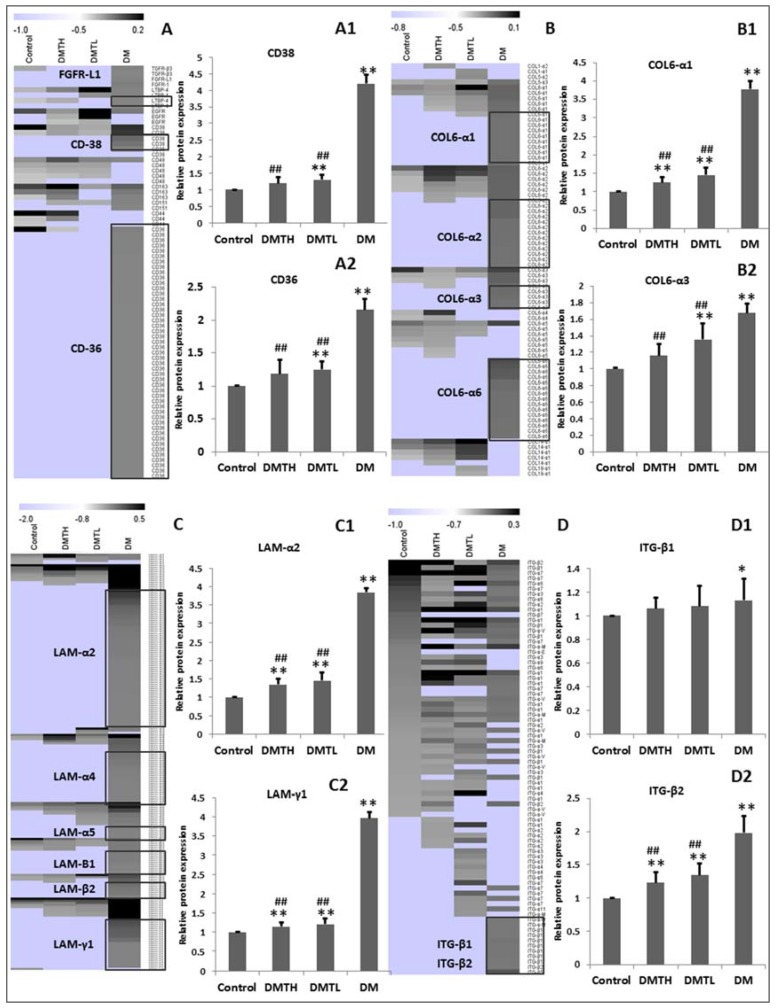
Expression differences in representative *N*-glycoproteins that can be significantly downregulated by 1-DNJ. **A**–**D**: CD38, CD36, COL-α1, COL-α3, LAM-α2, LAM-γ1, ITG-β1, and ITG-β2 expressional quantification, based on the peptide MS2 intensity analysis corresponding to **A1**, **A2**, **B1**, **B2**, **C1**, **C2**, **D1**, **D2**. ($\bar{X}\pm S.$ vs. Control: ** represents *p* < 0.01; * represents *p* < 0.05; vs. DM: ## represents *p* < 0.01; # represents *p* < 0.05). These results were analyzed with IBM-SPSS 19.0 software with one-way ANOVA method, and the supervised clustering presented with MeV-4.9.0 software.

Consistent with the expression patterns of serum biochemical markers in the four groups, the levels of some *N*-glycosylated proteins were downregulated by the intervention with 1-DNJ for six weeks. When comparing the treatment groups with the model group, the differences in the profiles of the DMTH group were much more significant than those of the DMTL group. Some glycosylated proteins are presented in Figure 6. It is noteworthy that the molecular functions of these glycoproteins were related to adhesion, hyperplasia, and fibrosis, such as integrin β [39,40], laminin [41], CD36, CD38 and collagen VI alpha (COL VI-α). The expression differences in these potential biomarkers prompted us to propose that the challenges of hyperglycaemia and glucose handling may be the internal factors that trigger and promote structural and functional changes in the myocardium.

### 2.6. FITC-Labelled Lectin Affinity Histochemistry

**Figure 7:**
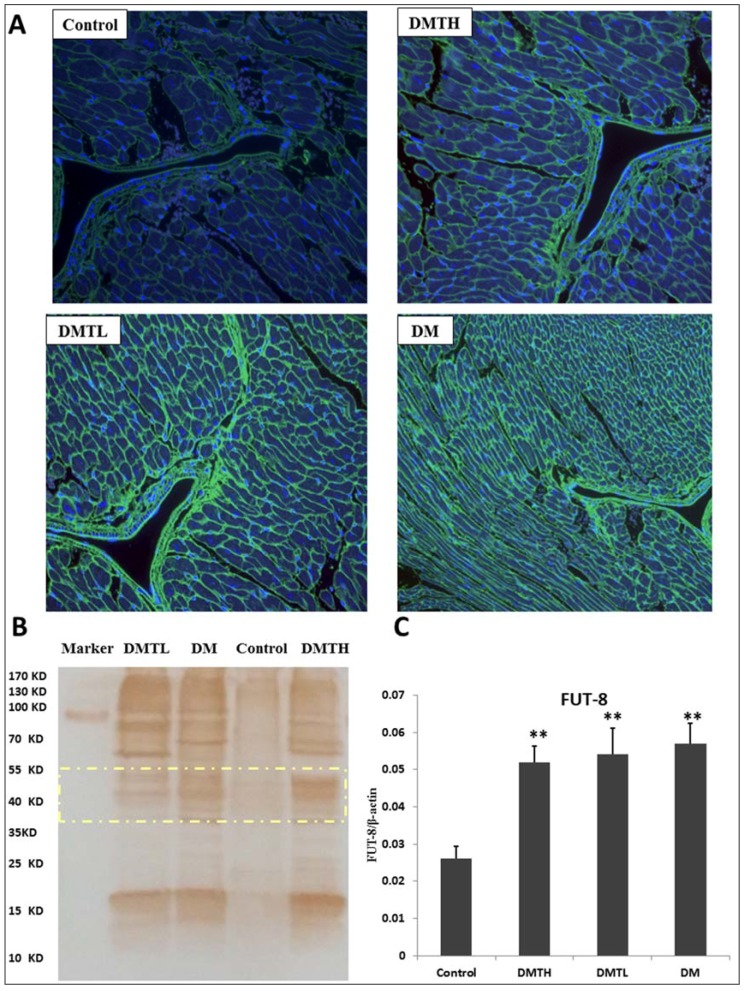
Lectin affinity fluorescence histochemical analysis of cardiomyocyte proteomic *N*-glycosylation. **A**: Images of FITC-labelled Lens culinaris agglutinin (LCA) lectin affinity fluorescence histopathology sections (×200) **B**: LCA lectin blot analysis. The yellow box represents the difference in the distribution of the mid-molecular weight *N*-glycosylated protein with α-1,6-fucose structure in the four groups. **C**: FUT8 mRNA expression measurement with ELISA kits. ($\bar{X}\pm S.$ vs. Control: ** represents *p* < 0.01. The statistical analysis was performed by one-way ANOVA with IBM-SPSS 19.0.

To determine whether the db/db mouse cardiomyocyte proteins were similar to the serum proteins that α-1,6-fucosylation increases significantly compared to the db/m mouse [24], we used Lens culinaris agglutinin (LCA) lectins to profile the glycan differences between the model group and the 1-DNJ-treated groups. LCA is an important tool for the study of glycoproteins with N-linked glycans. This lectin recognizes sequences containing α-linked mannose residues and also identifies additional sugars as part of the receptor structure. The α-linked fucose residue attached to the *N*-acetylchitobiose portion of the core oligosaccharide significantly enhances the binding affinity [42], and the resulting affinity is three times greater than that observed when an no α-linked fucose residue is attached to the core oligosaccharide [43,44]. Core fucosylation—the attachment of a fucose moiety to the innermost GlcNAc moiety of N-linked glycans with an α-1,6-glycosidic linkage—is a modification that is frequently found in natural and recombinant glycoproteins. Therefore, the main glycoproteins that are profiled by lectins are core α-1,6-fucose glycans. The lectin affinity histochemical analysis is presented in Figure 7. All the images of the sections showed that the extracellular and interstitial matrix were obviously stained. The glycoproteins with α-linked fucose structures were mainly distributed in the myocardium cytoplasmic membrane and intracellular stoma, as well as in serous membranes in the connective tissue. From the fluorescence intensity, we can see that the proteomic expression of α-1,6-fucosylated glycans in the DM group myocardium was upregulated significantly, and the coronary artery walls were clearly thickened compared with the control group. Moreover, some wrinkled cardiomyocytes can be seen in the section. Similarly, the DMTL group also presented with a strong fluorescence intensity in the ECM, and the difference between the control group and the DMTL group was also very significant. The fluorescence of the DMTH group was relatively weak, and the cell morphology was largely normal compared with that of the control group. 

### 2.7. Lectin Blot Analysis and *α*-*1*,*6*-Fucosyltransferase mRNA Expression Quantification

To verify the expression differences in N-linked glycoproteins with α-1,6-fucose structures obtained from lectin affinity histopathology analysis, lectin blot profiling was performed. Figure 7 indicates that the α-1,6-fucosylation levels in the DM, DMTH, and DMTL groups were all higher than that of the control group; however, the intergroup differences among these three groups were not significant, especially in the low molecular weight region and the high molecular weight region. From 40–55 kDa, the number of stained bands in the DMTH and DM groups was greater than that of the DMTL group, and the greyscale values calculated by ImageJ software indicated that in this region, the expressed α-1,6-fucosylated proteins were quantified as DMTH > DM > DMTL. In addition, the results of mRNA quantification by real-time fluorescence quantitative PCR showed that the α-1,6-fucosyltransferase FUT8 was expressed more strongly in the db/db mouse myocardium than in the db/m mice. Although the expression levels of the two treated groups were slightly lower than that of the model group, the intergroup differences were not significant. 

### 2.8. Transforming Growth Factor-β/Smad2/3 Pathway Protein Profiling with Western Blot Analysis

**Figure 8:**
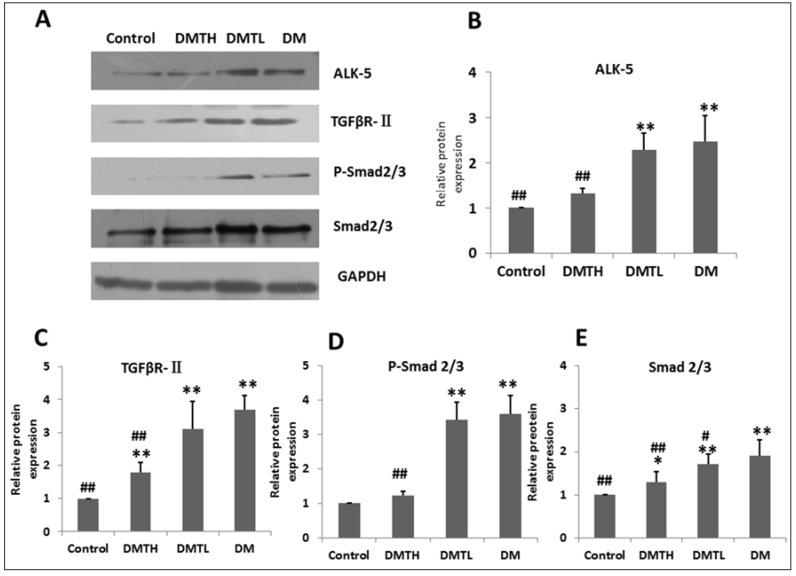
Semi-quantitative detection of TGF-β/Smad2/3 pathway protein expression via western blotting in the db/db mouse myocardium from four groups. **A** presents images from western blot analysis, which were developed with ECL kits and films. **B**–**E** represent comparisons of the semi-quantitative values of the four groups via one-way ANOVA of three independent measurements by IBM-SPSS 19.0. The values were obtained vs. GAPDH ($\bar{X}\pm S.$ vs. Control: ** represents *p* < 0.01; * represents *p* < 0.05; vs. DM: ## represents *p* < 0.01; # represents *p* < 0.05).

To verify the initial hypothesis of this study and explore the mechanism of the intervention effect of 1-DNJ on fibrosis, we profiled and quantified the cytokines in the TGF-β/Smad2/3 pathway by Western Blot (WB) analysis. The results are shown in Figure 8. From the images and calculations, we can see that the trends in the protein abundances of TGF-β/Smad2/3 pathway proteins in these four groups were similar. Compared with the control group, the expression levels of activing like kinase-5 (ALK-5) (namely, TβR-Ⅰ), TGFRβ-II, Smad2/3, and P-Smad2/3 in the db/db mouse myocardium were promoted significantly. 1-DNJ crude extract administration can inhibit the expression of these proteins, and compared with the DM group, the effects of the high-dose treatment were much more significant. This result indicated that the 1-DNJ crude extracts can be used to inhibit myocardium hypertrophy, ECM expansion, and myocardial fibrosis in DCM patients in the clinical setting.

## 3. Discussion

Protein glycosylation is one of the most prevalent post-translational modifications, and this modification can profoundly affect the intrinsic properties of a protein. The oligosaccharides of glycoproteins can regulate the recognition process directly, such as through signalling, cell adhesion, immune responses, and host–pathogen interactions. If subtle changes in glycoproteins occur, significant biological functions will be affected. 

The relationship between protein-aberrant *N*-glycosylation and tissue fibrosis is complicated. Analysis of sputum from patients with cystic pulmonary fibrosis indicated that *N*-glycans were terminal α2,6-sialylated, α-1,6-core fucosylated, β1,4-bisecting GlcNAcylated, and compositions were pauci-mannosylated [45]. Similarly, the study of modifications of human total serum *N*-glycome during liver fibrosis-cirrhosis showed that the core fucosylation increased obviously after the depletion of IgG and IgA from serum [46]. In addition, the TGF-β receptor core fucosylation was closely related to final fibrosis [25]. In the case of myocardial fibrosis, much more research was concerned with the *O*–GlcNAc modification [47], meanwhile, the aberrant *N*-glycosylated modification reports were scarce. However, it is difficult to deny that some biomarkers of cardiac fibrosis, such as galectin-3 [48] and CTGF [49], were all *N*-glycoproteins.

In essence, fibrosis is the tissue repair response that protects the tissue integrity after damage. However, if this repair is overactive, too strong, or uncontrolled, it can cause fibrosis and lead to a functional decline in the organ. Hyperglycemia causes oxidative stress, increased production of AGEs, endothelial dysfunction, disorders of coagulation and changes in fibrinolytic function. In the case of myocardial cells, the repeated and sustained damage described above will induce a large amount of interstitial fibrous connective tissue (ECM) to repair the defective tissue—that is, the pathological changes of fibrosis will occur. The histopathological analysis in this study suggests that even in the primary stage of diabetes, changes in the physiological structure of the db/db mouse myocardium have occurred. This result was verified by the expression levels of the hyperplasia- and fibrosis-related cytokines CTGF and hydroxyproline. In this study, we sought to improve our understanding of the intervention effect of 1-DNJ on fibrosis. Many reports have demonstrated that the key cytokine TGF-β1 plays a crucial role in the process of myocardial fibrosis. During the TGF-β/Smads signalling processes, the important post-translational modification of TβRII is the core fucosylation of the *N*-glycan. In other words, these reactions in this pathway will be blocked if this core fucosylation does not happen [50]. Our western blot analysis showed that TβRI, TβRII, and R-Smads are all overexpressed in the db/db mouse myocardium, and this result was consistent with the results of lectin histochemistry and lectin blot profiling.

Since the core α-1,6-fucosylation of the myocardium was downregulated by 1-DNJ administration in db/db mice, α-1,6-fucosyltransferase (FUT8) expression levels were detected in these four groups. We used real-time fluorescence quantitative PCR to profile FUT8 mRNA expression. The level of α-1,6-fucosyltransferase mRNA was increased in the myocardium of the db/db mice. Although the expression levels of the 1-DNJ administered groups were slightly lower than that of the model group, the intergroup differences were not significant. This result illustrated that 1-DNJ had no significant effect on the expression of FUT8. This observation also indirectly showed that 1-DNJ-mediated inhibition of core α-1,6-fucosylation is likely to be related to effects on the substrate concentration. N-linked glycan synthesis is known to start in the endoplasmic reticulum (ER) and end in the Golgi apparatus [51]. First, the dolichol-bound oligosaccharide precursor Glc3Man9GlcNAc2 is transferred to Asn residues on nascent polypeptides by an oligosaccharyltransferase (ost); then, the oligosaccharide undergoes trimming of the glucose residues and some mannose residues, first in the ER and then in the Golgi, followed by the addition of branching *N*-GlcNAc and additional sugars—such as galactose, fucose, and sialic acid—by Golgi glycosyltransferases, to form hybrid and complex *N*-glycans. Although FUT8 is the key enzyme for core fucosylation formation, it cannot directly glycosylate full-size glycans, and can only accept an *N*-glycan core with a free GlcNAc residue at the α-1,3-mannose branch as its substrate [52]. It has been reported that N-linked complex oligosaccharide formation can be inhibited by 1-DNJ; however, the mechanism by which 1-DNJ exerts inhibitory effects on core α-1,6-fucosylation should be explored in further studies.

The purified 1-DNJ contains some flavonoids, such as rutin, quercetin, and kaempferol in this study. Rutin has efficacy in improving post-thrombotic syndrome [53], venous insufficiency [54], or endothelial dysfunction [55]; however, its low bioavailability limits its application. Quercetin has been reported to inhibit the oxidation of other molecules, and hence is classified as an antioxidant [56], similarly, the bioavailability of quercetin in humans is low and highly variable (0–50%), and its rapid excretion also limits its medical usages [57]. Kaempferol has a wide range of physiological activities, such as anti-oxidant and anti-inflammation, unfortunately, its histocompatibility was not so good. As a matter of fact, the concentration of the flavonoids in 1-DNJ extract was extremely low (validation results are in the Supplementary File 1. In this experiment, we released alkaloids from the resin column with 0.5 M ammonia solution. Some salt solutions with strong elution capabilities, such as 5–10% sodium chloride, were not used during the release of alkaloids. Similarly, organic reagents like ethanol, acetonitrile, or methanol were not used in the elution, either. Owing to the effect of charge redistribution, these reagents will easily elute some impurities, such as flavonoids, from the strong cation exchange resins in the column. Based on the above discussion, we believe that the effects of flavonoids in 1-DNJ extract on experimental animals are extremely weak, and 1-DNJ is the main substance that inhibits myocardial fibrosis in db/db mice.

## 4. Materials and Methods

### 4.1. Reagents and Drugs

BB was prepared by our team. Insects of *Bombyx mori* L. were purchased from the Sericulture Institute of the Academy of Agricultural Sciences (Sichuan, China), and mulberry leaves were purchased from the mulberry planting base in Huzhou City in Zhejiang Province. The fungus *Beauveria bassiana* was purchased from the China General Microbiological Culture Collection Center (CGMCC). After the expansion culture of *B. bassiana*, the generated spores were washed with physiological saline and sprayed on the surface of silkworms. The preparation was made under the conditions of 95% relative moisture and 26–28 °C. The silkworms were infected with the fungi and then became stiff, until the bodies were wrapped with the white mycelium. The stiff silkworms were collected and dried in an electric blast-drying oven at 30 °C.

Trypsin and PNGaseF (PNGF) were obtained from New England Biolabs (Ipswich, MA, USA). The chemical reagents methanol, acetonitrile, iodoacetamide (IAA), 1,4-dithiothreitol (DTT), trifluoroacetate (TFA), and formic acid (FA) were obtained from Sigma-Aldrich (Shanghai, China). Ammonium bicarbonate (ABC) and urea were obtained from Bio Basic Inc. (Markham, ON, Canada). The standards of 1-DNJ, kaempferol, quercetin and rutin were purchased from Aladdin Reagents (Shanghai, China). Strong cation exchange resins and other chemicals and reagents were obtained from Sinopharm Chemical Reagent Co., Ltd. (Beijing, China). Hydrophilic chromatography beads (5 μm, 120 Å) were obtained from Agela Technologies (Tianjin, China). The LCA-FITC reagent was obtained from Vector Labs (Burlingame, CA, USA). Antibodies were purchased from Abcam (Cambridge, UK). 

### 4.2. 1-Deoxynojirimycin Extract Preparation and Purity Detection 

The drugs were ground into a powder and then refluxed with distilled water (1:10, *w*/*v*) for 1 h. The filtrates were collected, and the residues were refluxed in water (1:10, *w*/*v*) for another 0.5 h. Two batches of filtrates were combined. Afterwards, the extract was precipitated by adding enough ethanol to generate a final 75% concentration. The supernatant was obtained by centrifugation (5000 rpm, 20 min). The ethanol was removed, and the residual liquid was adsorbed by 732-H^+^ cation ion exchange resins. The resins were washed with deionized water three times, and the 1-DNJ was eluted with 10 times the column volume of a 0.5 M ammonia solution. The elution was lyophilized, and the 1-DNJ was reconstituted with methanol. With the use of an evaporative light scattering detector (ELSD) and an LC-NH2 column (4 × 250 mm, 5 μm; Elite, Dalian, China), detection was conducted with a system of acetonitrile and water (80:20) as the mobile phase, and a flow rate of 0.8 mL/min. The column temperature was 40 °C, and the vaporizer temperature was 100 °C. The nitrogen flow rate was set as 2.3–2.5 standard liter per minute (SLPM). Other chemicals were determined with an HPLC system with the UV detector at 360 nm. The analysis column was an ODS-C18 column (4.6 × 250 mm, 5 μm; Elite), and the mobile phase consisted of 0.1% acetic acid (A, SIGMA-ALDRICH, St. Louis, MO, USA) and acetonitrile (B, SIGMA-ALDRICH, St. Louis, MO, USA), with the gradient elution mode as follows: 0.01–20.00 min, B: 30–85%, A: 70–15%; 20.01–30.00 min, B: 85–85%, A:15–15%; and 30.01–35.00 min, B: 85–30%, A:15–70%.

### 4.3. Animals and Administration

Approximately five-week-old C57BLKS/db/m and C57BLKS/db/db mice were purchased from CAVENS Lab Animal Ltd. (Changzhou, China). The animals were maintained in a room with a controlled temperature (22 ± 2 °C) and a 12-h light/dark cycle with free access to food and water. The animal use protocol has been reviewed and approved by the Animal Ethical and Welfare Committee (AEWC) of Hebei University, the approval date was January 20, 2018. The approval number was 2018001. All the animals were cared for in accordance with the Regulations of Experimental Animal Administration issued by the State Committee of Science and Technology of the People’s Republic of China on 14 November 1988. Every group of mice consisted of four males and four females. The db/m mice in the control group (Control) were treated orally with sterile water instead of the drug, and the db/db mice in the DMTL group were treated with 5.0 mg/kg 1-DNJ orally. The db/db mice in the DMTH group were treated with 1-DNJ at a dose of 10.0 mg/kg. The dose was established based on the published literature [58]. The model group (DM) consisted of db/db mice treated with 0.2 mL of sterile water. This administration procedure was continued for six weeks.

### 4.4. Clinical Biochemistry Indicator Determination and Histopathology Analysis

Before blood collection, all mice were fasted for 12 h, and their water drinking was controlled in moderation. The animals were anaesthetized with pentobarbital sodium (120 mg/kg). When the animals were ataxic and the righting reflexes disappeared, the blood was sampled immediately. After centrifugation (3000 rpm, 15 min), the key biochemical indicators, including FBG, TC, TG, HbA1, and AGEs, were assessed with ELISA kits (Beyotime, Beijing, China). The animals were all euthanized, and the hearts were removed from the chests immediately and washed with precooled PBS. Half of the heart tissue was stored in liquid nitrogen, and the other half was preserved in a formalin solution. After fixation, the tissues were sliced into histopathological sections, and these sections were stained with HE- or FITC-labelled LCA lectins (1:2000; Vector Labs, Burlingame, CA, USA). Finally, the sections were observed under a microscope. 

For the HE staining, the number of myocardial necrosis, micrometabolic necrosis, and myocardium scar lesions were counted in the high magnification field of view, with 20–30 visual fields per case. In a 200-microscopic view of the microscope, micrometer-sized microarterial wall thickness was mapped using a 1 cm long line. Five fibrosis spots were selected for each slice with the help of high magnification microscope field. Images in JPEG format were taken, and integrated optical densitometry was performed on the samples using professional medical analysis measurement image software (Image Pro-Plus V5.2, Media Cybernetics, Rockville, MD, USA).

For the fluorescent immunohistochemistry, the true-color fluorescence images were obtained by using an Olympus IX70 (Olympus, Tokyo, Japan) epifluorescence microscope, equipped with a long-pass filter and a digital color CCD camera (Olympus). Fluorescence spectroscopy was carried out by attaching a spectrometer to the microscope side port. At the same magnifications (e.g., 200×), we measured the fluorescence intensity from a series of samples with the highest α-1,6-fucosylated protein expression. This intensity was set as 100%, and was used for further calculations. To ensure statistical validity, multiple spectra (30–50) of representative regions were taken and averaged in the analysis.

### 4.5. Protein Extract and Digestion

Approximately 100 mg of frozen tissue, which was ground into powder with liquid nitrogen assistant, was used to extract proteins with 500 μL of lysis buffer (10 mM Tris-HCl with 4% SDS and 1% protease inhibitor, pH = 8.0). With the aid of a sonicator, the proteins were extracted completely, and the concentration was determined with the Bradford method [59]. Half of the extract was used for ELISA kit analysis, and the rest was used for mass spectrometry-based shotgun proteomic analysis. Approximately 500 μg of protein was denatured by heating at 95 °C for 10 min, and then digested into peptides according with the filter aided proteome preparation (FASP) method [60].

### 4.6. Glycosylated Peptides Enrichment and N-Glycan Resection

The glycosylated peptides were enriched through solid-phase extraction with hydrophilic chromatography, and the *N*-glycans were removed from the peptides with the endoglycosidase PNGF. All procedures were carried out as described in a previous publication from our team [41]. After lyophilization, all the peptides were reconstituted with 0.5% FA, and the concentration was detected with a Nano-drop 2000 (Thermo Fisher Scientific, Waltham, MA, USA). 

### 4.7. Proteomic Analysis with LC-MS/MS

Approximately 700 ng of peptides was analysed by nano-LC-MS/MS. The samples were separated with an Easy-1000 nano-liquid phase system (Thermo Scientific, Waltham, MA, USA); mobile phase A was 0.5% formic acid, and mobile phase B was a mixture of acetonitrile (ACN) and 0.5% FA at a volume ratio of 98:2. The flow rate was 300 nL/min, and the B phase gradient rose from 5% to 50% over 60 min. A Q-Exactive mass spectrometer (Thermo Scientific, Waltham, MA, USA) was used with positive ion mode scanning. The scan range was 300–1400 *m*/*z*, and the resolution was 7000. Secondary mass spectrometry (DDA) was performed for the data dependence model. The high-energy collision dissociation (HCD) fracture energy was 27, the select primary atlas 75 ion signal was strongest in secondary mass spectrum scanning, and the time was set to 18 s to rule out dynamic changes.

### 4.8. Database Retrieval Conditions and Statistical Analysis Toolkits

Proteomic Discovery 1.3 series software was used to change the extensions of raw files to mgf files, as the operation manual described. The mgf files were loaded in pFind 3.1.0 software (http://pfind.ict.ac.cn/), and the total protein database search conditions were set as follows: the mass spectrum level accurate mass number deviation was 20 ppm or less, and the secondary search quality deviation was 15 mmu. For the trypsin digestion type, the fixed modification was cysteine urea methylation, and the variable modifiers were as follows: methionine oxidation, protein N-term acetylation, and asparagine deamination, with a maximum allowed missing cleavage site number of 2. The database for searching matched proteins through peptide sequence and modification information was Ref_mouse_20161201. The analysis toolkits included pFind software (pFind Studio, Beijing, China), IBM-SPSS 19.0 (IBM, Chicago, IL, USA), MeV-4.9.0 (J. Craig Venter Institute, Rockville, MD, USA), and DAVID (https://david.ncifcrf.gov/) functional annotation in this study. The protein accessions obtained from searching were changed into the corresponding gene symbols, and the protein intensions were used to complete the cluster analysis. Comparison of multiple groups of sample means was conducted with a completely randomized one-way analysis of variance by IBM-SPSS 19.0.

### 4.9. Lectin Blot Analysis

The samples for concentration detection were denatured at 95 °C for 10 min. Then, aliquots of approximately 20 µL were separated with one-dimensional SDS-PAGE. The proteins were transferred to PVDF membranes (Merck & Co Inc, Kenilworth, NJ, USA) at 120 volts for 60 min in an ice bath. The PVDF membranes were blocked with 5% bovine serum albumin (BSA). During every wash step, the membrane was rinsed gently three times. Then, the membrane was incubated with biotinylated LCA lectins at 4 °C overnight. After three washes, the membrane was developed with an avidin-labelled DBA developing kit (CWBio, Beijing, China). Finally, the bands were imaged with ImageJ-1.49 software (National Institute of Mental Health, Bethesda, MD, USA). The image type was changed into gray-white and the background effects were eliminated. The measurements and scales of area, intensity, and grayscale value were set moderately. 

### 4.10. Western-Blot Analysis

As described for the lectin blot procedures, the PVDF membranes were blocked with 5% BSA and then incubated with primary antibodies overnight at 4 °C. After three washes, the membrane was incubated with a horseradish peroxidase-conjugated secondary antibody at room temperature for 1 h. Then, the PVDF membrane was washed three times. The protein bands from the four groups of samples were detected using an ECL kit (CWBio, Beijing, China), and protein expression levels were quantified using ImageJ analysis software.

### 4.11. Real Time PCR Analysis of FUT8

FUT8 mRNA expression was quantified with real-time fluorescence quantitative PCR, using the SYBR Primer Script RT-PCR Kit (Takara, Otsu, Shiga, Japan), according to the manufacturer’s protocol. The primers were synthesized by BGI Genomics (Shenzhen, China), as follows. For FUT8, the forward primer (from 5′ to 3′) sequence was GCTACCGATGACCCTGCTTTG, and the reverse primer sequence was CCGATTGTGTAATCCAGCTGAC. For GAPDH, the forward primer sequence (from 5′ to 3′) was GCACCGTCAAGGCTGAGAAC, and the reverse primer sequence was TGGTGAAGACGCCAGTGGA. The levels of gene expression were calculated with the ΔΔ*Ct* method after normalization to GAPDH, and all samples were analysed in triplicate.

## 5. Conclusions

The pathological changes of cardiomyocyte apoptosis, stripe disappearance, and scar formation occurred in the myocardial tissue of db/db mice, and the expression levels of cytokines related to fibrosis were upregulated significantly. The alkaloid 1-DNJ can effectively reduce the N-linked glycosylation level of myocardium proteins and inhibit cardiomyocyte apoptosis and scar formation. The α-1,6-fucose glycosylation levels in the db/db mouse myocardium were also reduced by 1-DNJ. The TGF-β/Smad2/3 pathway, which is closely related to myocardial fibrosis, was also impaired by 1-DNJ administration, and this result verified the results from LCA lectin affinity histochemical analysis, as well as lectin blot analysis of the α-linked fucosylation of the *N*-glycoproteins expressed in the left ventricular myocardium. However, 1-DNJ had no significant effect on the expression of FUT8. From the perspective of *N*-oligosaccharide formation, it can be speculated that the 1-DNJ crude extract from BB delays glucose production by inhibiting glucosidase activity to interrupt the process of glucose-mediated attack on cardiomyocytes. Therefore, the mechanism of 1-DNJ to relieve DCM-associated fibrosis can be concluded to be the inhibition of *N*-GlcNAc formation and the reduction of substrate concentration.

## Supplementary Materials

Supplementary materials can be accessed at: http://www.mdpi.com/1422-0067/19/6/1699/s1.
