# Supplementary material for: A Crude 1-DNJ Extract from Home Made Bombyx Batryticatus Inhibits Diabetic Cardiomyopathy-Associated Fibrosis in db/db Mice and Reduces Protein N-Glycosylation Levels
Source: Int J Mol Sci. 2018 Jun 7;19(6):1699. doi: 10.3390/ijms19061699 (PMC6032278; doi:10.3390/ijms19061699)
Supplement: Supplementary file 1 [file ijms-19-01699-s001.zip › Supplementary File 1.pptx]

## Slide 1
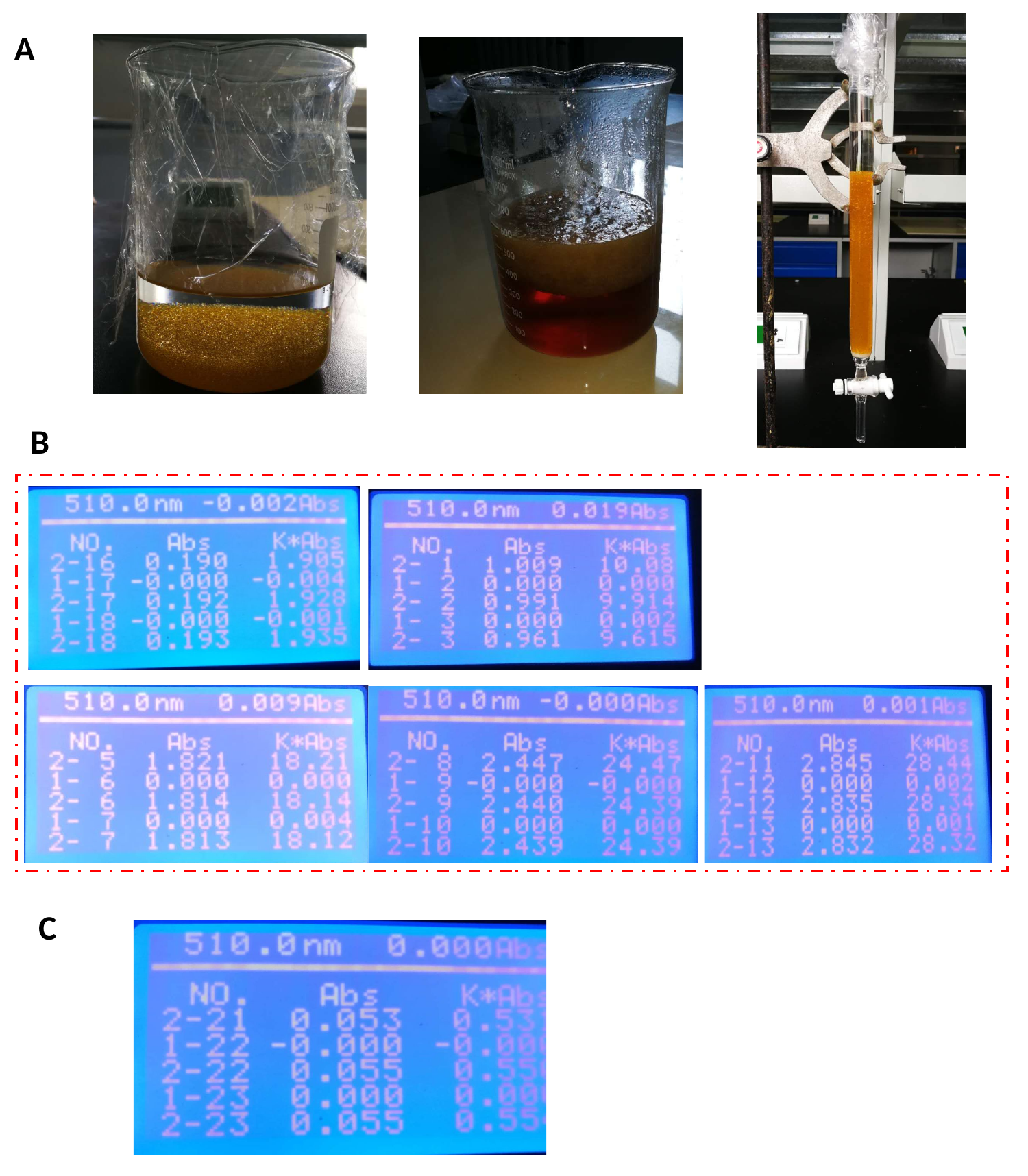

A
B
C

## Slide 2
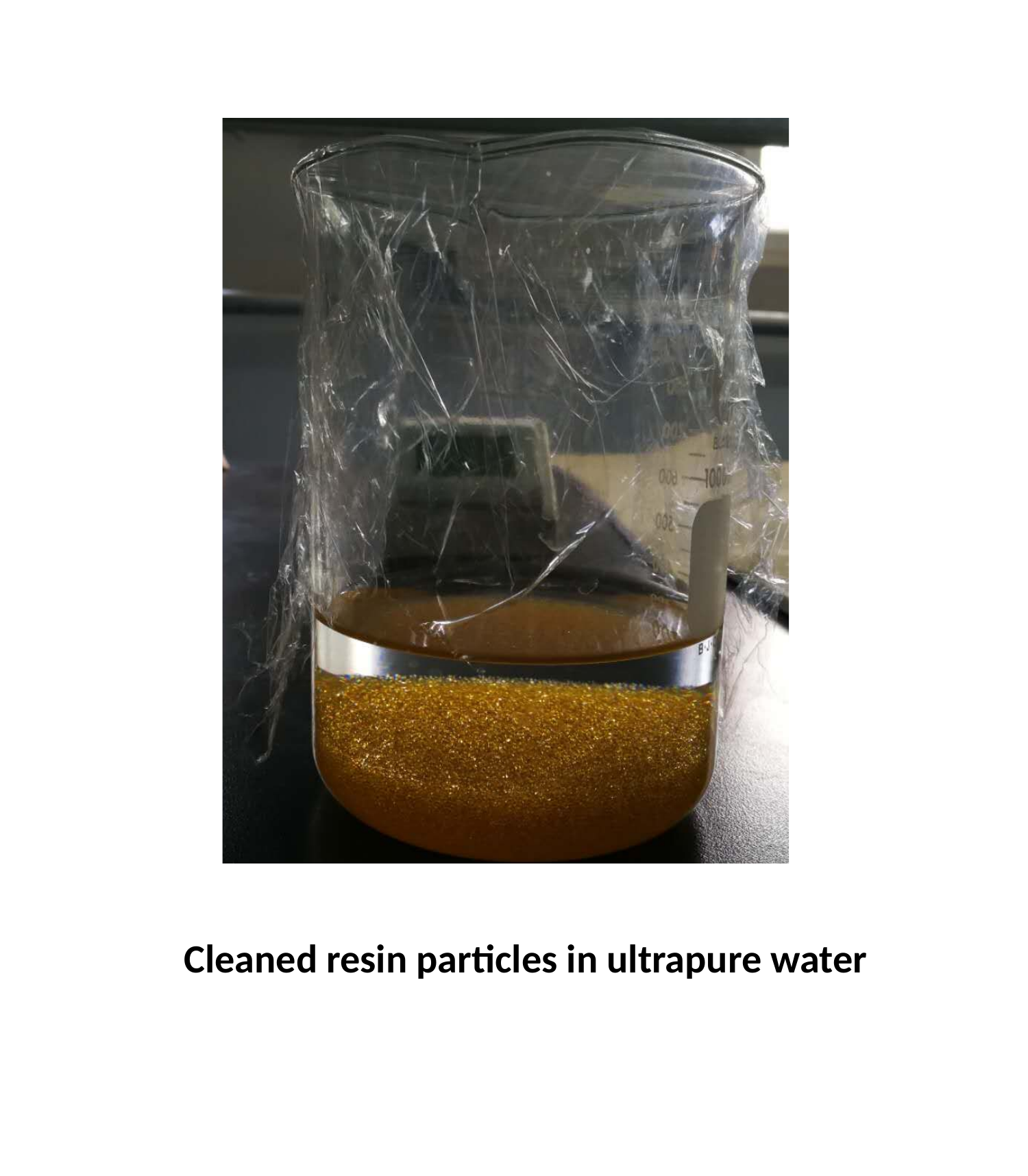

Cleaned resin particles in ultrapure water

## Slide 3
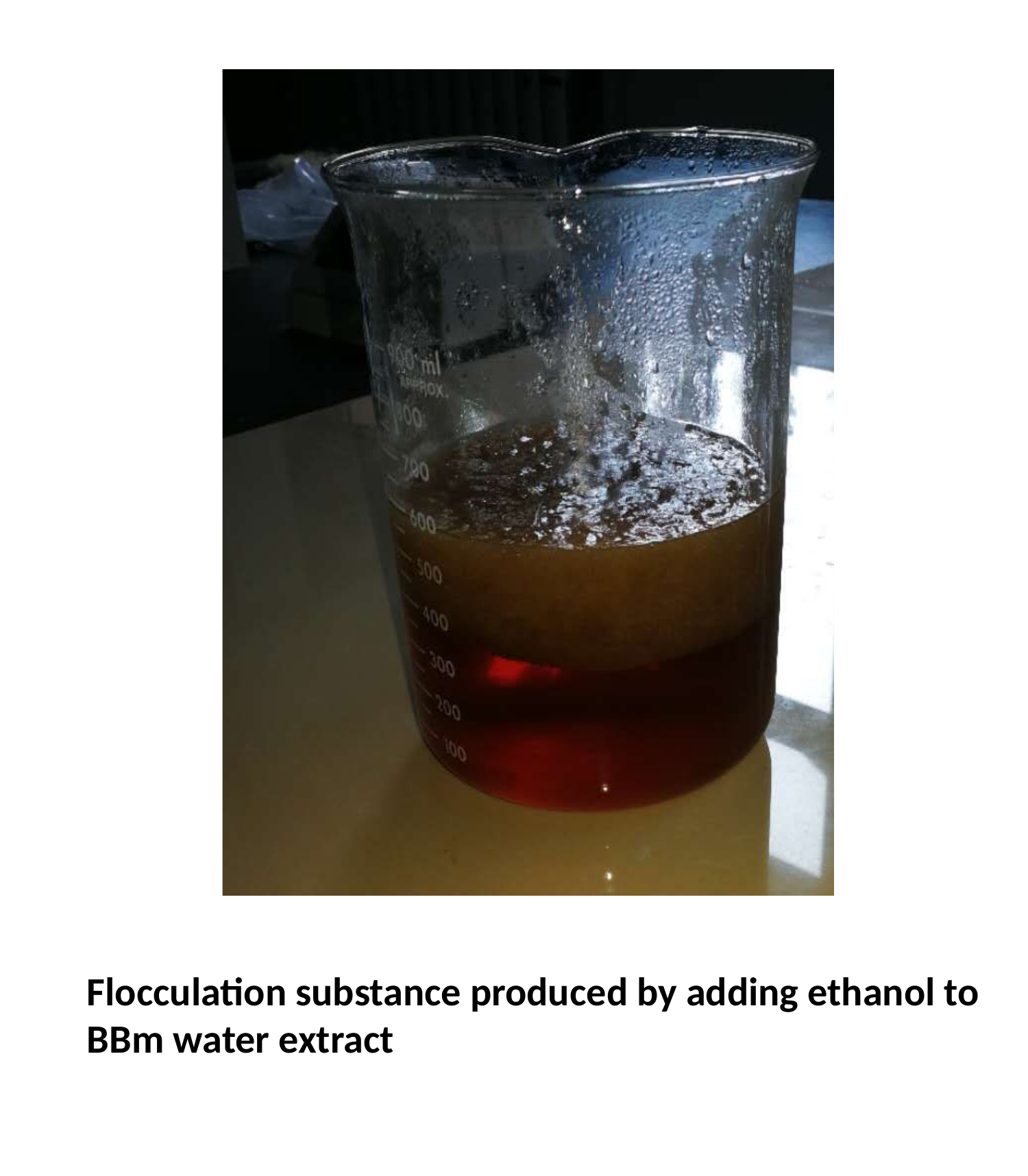

Flocculation substance produced by adding ethanol to BBm water extract

## Slide 4
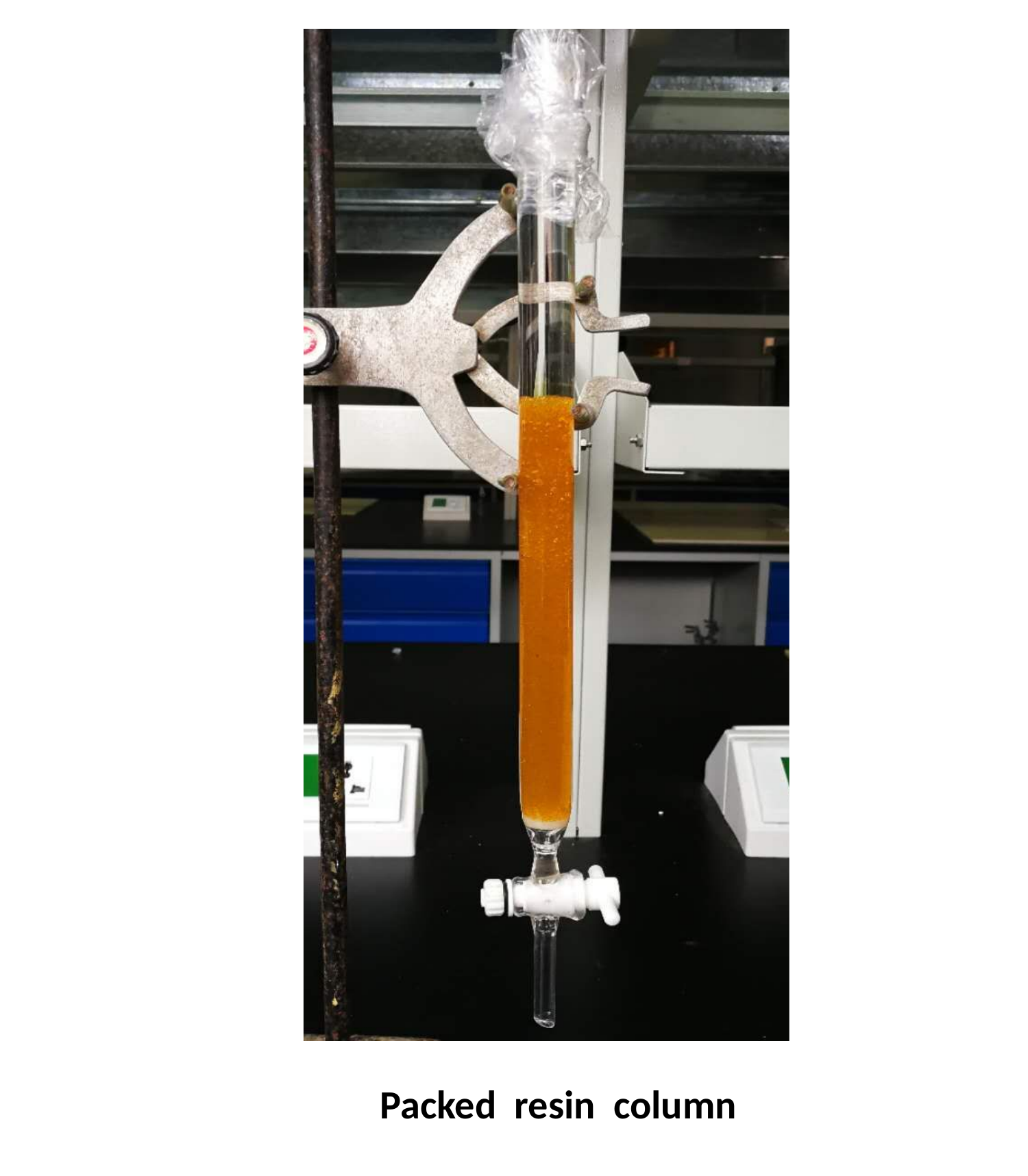

Packed resin column
