# Supplementary material for: A Crude 1-DNJ Extract from Home Made Bombyx Batryticatus Inhibits Diabetic Cardiomyopathy-Associated Fibrosis in db/db Mice and Reduces Protein N-Glycosylation Levels
Source: Int J Mol Sci. 2018 Jun 7;19(6):1699. doi: 10.3390/ijms19061699 (PMC6032278; doi:10.3390/ijms19061699)
Supplement: Supplementary file 1 [file ijms-19-01699-s001.zip › Supplementary File 2.pdf]

International Journal of
Molecular Sciences

MDPI

Determination of Total Flavonoids in 1-DNJ Enrichment Liquid by Ultraviolet Spectrophotometry

1. 001 × 7 (732) type strong cation ion exchange resin treatment

Took 50g of resin particles (moisture content is 45-55%) and placed them in a clean beaker. Firstly, the resin was washed with ultrapure water. During the washing process, it was necessary to stir the resin gently with a glass rod, and then poured the liquid. The resin particles were washed for a total six times.

Next, the resin was soaked in with 2 times the volume of resin in 5% hydrochloric acid. 30 minutes later, the resin was rinsed with ultrapure water until the pH of the eluent was around 6.

After that, the resin was soaked in with 2 times the volume of resin in 5% sodium hydroxide. 30 minutes later, the resin was rinsed with ultrapure water until the pH of the eluent was around 9.

Then, the resin was soaked in 5% hydrochloric acid again to convert it into H-type. The volume of hydrochloric acid was 3 times more than the resin volume. The soaking time was no less than 2 hours.

The treated resin was rinsed with ultrapure water again and again, until the pH of the eluent was 7, then the column can be packed.

2. 1-DNJ extract preparation

The white stiff silkworm was placed in an electric blast oven at 60°C to dry completely. After 2 h, the drug was taken out. When the drug got cool, 80g of silkworms were grinded in to powder with a grinder. It was necessary to make sure that all of the powder passed through a 40-mesh sieve. The powder was placed in a 2000 ml round-bottomed flask and 1000 ml of distilled water was added. The flask was boiled in boiling water bath and the reflow extraction was last for 1 hour. After that, the extract was filtered with a suction filter. Then, 1000 ml of distilled water was added to the residue and the mixture was boiled again. The second reflow extraction was ended after 1 hour. The flask was taken out, and the extract was filtrated. The twice filtrate was combined and the total volume was recorded.

Next, enough ethanol was slowly added to the filtrate with stirring to produce a large amount of flocculent and made the final ethanol concentration to 75%. The extract was left in 4°C overnight. Then, the precipitate was removed by suction filtration, and the resulting filtrate was placed in a vacuum rotary evaporator to recover ethanol. The residual water solution was taken out and concentrated to 800ml. Next, 300 ml of the solution was taken out and volume was reduced to 50 ml by a water bath concentration. After that, 1.05 ml hydrochloric acid with a concentration of 37% was added in the 50ml solution to make the final concentration of hydrochloric acid in the liquid is 1%. Accurately absorbed 20 ml of the acidified liquid and placed it in the packed strong cation exchange resin column. Let the resin particles statically adsorb for two hours, and then loosen the glass plug's live plug to make the flow rate was at 3 ml/min. Afer that, about 6 times (300 ml) column volume of ultrapure water was used to wash resin column. After washing, about 4 column volumes (200 ml) of 0.5 moles of ammonia was used to release the alkaloids from the ion column, When the elution was ended, the eluent was heated with a water bath in fume hood to remove the ammonia. Finally, the residue was concentrated and made a final 50 ml volume.

3. UV-visible spectrophotometric determination of total flavonoids in eluent

3.1. The drawing of the standard curve:

1.0 ml rutin solutions with different concentrations were put in 10.0 ml volumetric flasks respectively. After that, 0.4 ml 5% sodium nitrite solution was added. The volumetric flasks were shook for 6 min gently. After that, 0.4 ml 10% aluminum nitrate solution was added, and the

volumetric flasks were shook for another 6 min gently. Next, 4 ml 4% hydroxide sodium solution was added, and the reaction was last for 15 min. Finally, made the solutions in different flasks to a final volume as 10ml. Measured the absorbance of all solutions at 510 nm. The rutin concentration was as abscissa and absorbance was as ordinate, prepared the standard curve. The results were shown in the table-1. The standard curve was shown in figure-1.

Tabel 1. Rutin solutions absorbance determination .

Rutin concentration (mg/ml)	Abs1	Abs2	Abs3	N	average
0	0	0	0	3	0
0.00673	0.19	0.192	0.193	3	0.192
0.0971	1.009	0.991	0.961	3	0.987
0.1942	1.821	1.814	1.813	3	1.816
0.2913	2.447	2.44	2.439	3	2.442
0.3884	2.845	2.835	2.832	3	2.837

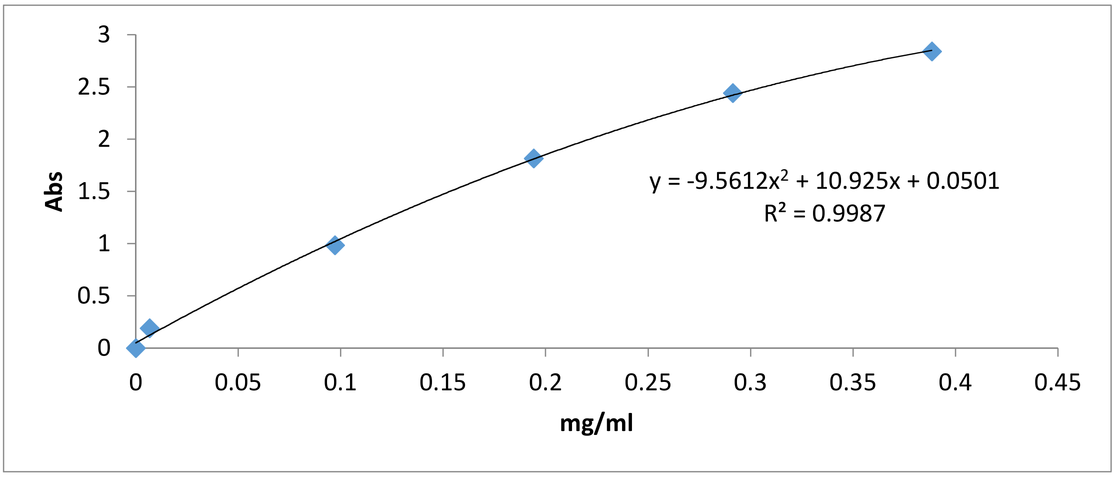

Figure 1. Standard curve of rutin with concentration and absorbance in 510nm.

3.2. Determination of total flavonoids in the eluent of the extract

Similarly, accurately draw 1 ml solution from the 50 ml eluent into a 10 mL volumetric flask and measure the absorbance at 510 nm as described above. The results were shown in table-2.

Table 2. Total flavonoids absorbance determination in sample.

Flavonoids concentration (mg/ml)	Abs1	Abs2	Abs3	N	average
X	0.053	0.055	0.055	3	0.054

From the results in table-2, we can calculate the total flavonoid concentration in the extract eluent with the curve equation, and the X (concentration) was 0.00036mg/ml. That means, 50 ml eluent contained 0.18mg total flavonoids, which equivalent obtained from 12g *Bombyx Batryticatus* mori.L. correspondingly (80g×3÷8×2÷5=12g).

Our results showed that the total flavonoids in the 1-DNJ extract is extremely low just as we reported in the manuscript.

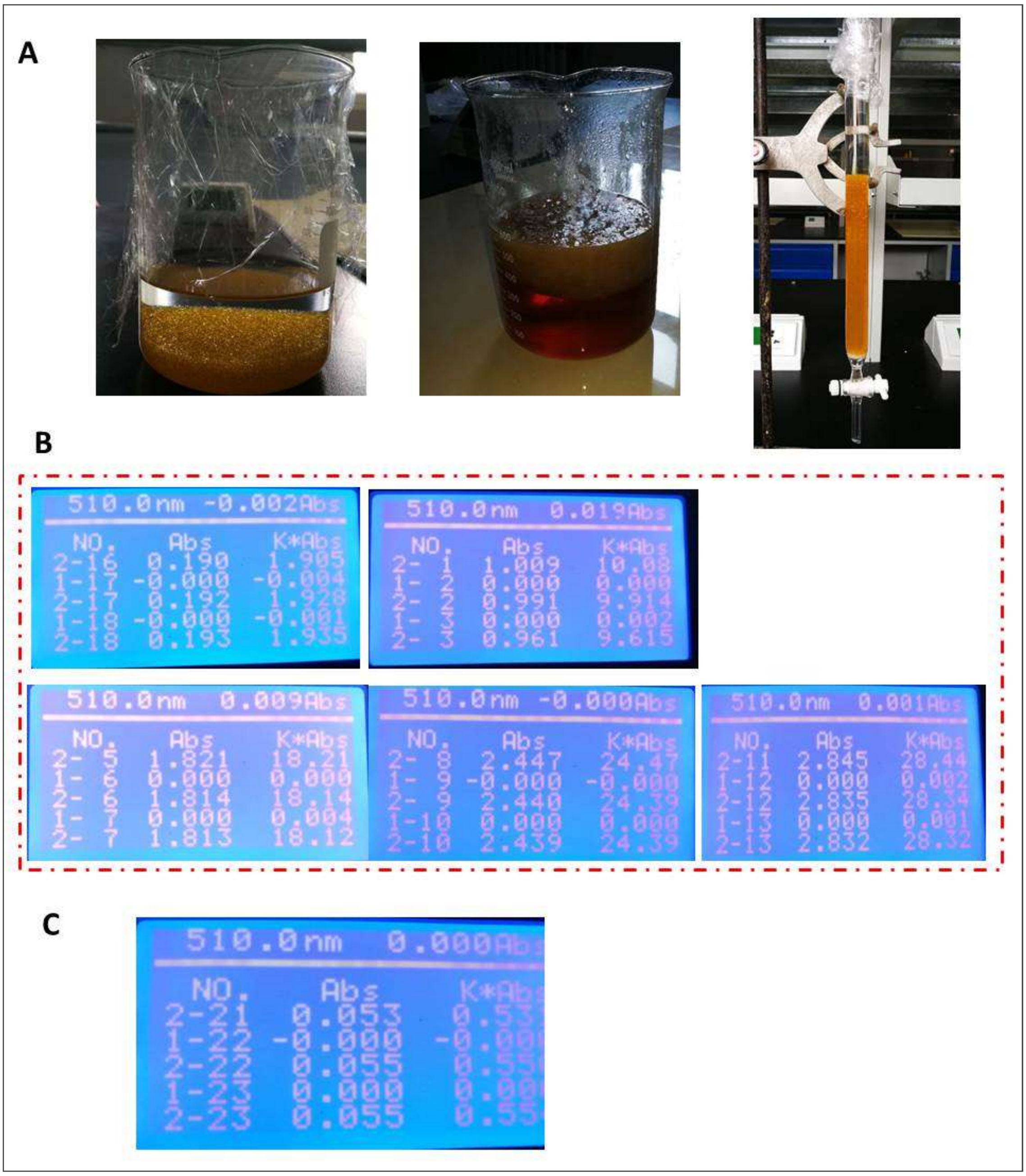

Figure 2. Descriptive results of the experimental process presented graphically. **A.** washed resin particles and packed column. **B**. Absorbance of different concentration rultin solutions. **C**. The absorbance of flavonoids in extract solution from eluent measured in 510nm.
